# A systematic review and meta-analysis on herpes zoster and the risk of cardiac and cerebrovascular events

**DOI:** 10.1371/journal.pone.0181565

**Published:** 2017-07-27

**Authors:** Nathaniel Erskine, Hoang Tran, Leonard Levin, Christine Ulbricht, Joyce Fingeroth, Catarina Kiefe, Robert J. Goldberg, Sonal Singh

**Affiliations:** 1 Department of Quantitative Health Sciences, University of Massachusetts Medical School, Worcester, Massachusetts, United States of America; 2 Lamar Soutter Library, University of Massachusetts Medical School, Worcester, Massachusetts, United States of America; 3 Division of Infectious Diseases and Immunology, Department of Medicine, University of Massachusetts Medical School, Worcester, Massachusetts, United States of America; 4 Division of General Internal Medicine, Department of Medicine, University of Massachusetts Medical School, Worcester, Massachusetts, United States of America; 5 Department of Family Medicine and Community Health, University of Massachusetts Medical School, Worcester, Massachusetts, United States of America; Southern Illinois University School of Medicine, UNITED STATES

## Abstract

**Background:**

Patients who develop herpes zoster or herpes zoster ophthalmicus may be at risk for cerebrovascular and cardiac complications. We systematically reviewed the published literature to determine the association between herpes zoster and its subtypes with the occurrence of cerebrovascular and cardiac events.

**Methods/Results:**

Systematic searches of PubMed (MEDLINE), SCOPUS (Embase) and Google Scholar were performed in December 2016. Eligible studies were cohort, case-control, and self-controlled case-series examining the association between herpes zoster or subtypes of herpes zoster with the occurrence of cerebrovascular and cardiac events including stroke, transient ischemic attack, coronary heart disease, and myocardial infarction. Data on the occurrence of the examined events were abstracted. Odds ratios and their accompanying confidence intervals were estimated using random and fixed effects models with statistical heterogeneity estimated with the I^2^ statistic. Twelve studies examining 7.9 million patients up to 28 years after the onset of herpes zoster met our pre-defined eligibility criteria. Random and fixed effects meta-analyses showed that herpes zoster, type unspecified, and herpes zoster ophthalmicus were associated with a significantly increased risk of cerebrovascular events, without any evidence of statistical heterogeneity. Our meta-analysis also found a significantly increased risk of cardiac events associated with herpes zoster, type unspecified.

**Conclusions:**

Our results are consistent with the accumulating body of evidence that herpes zoster and herpes zoster ophthalmicus are significantly associated with cerebrovascular and cardiovascular events.

## Introduction

Approximately 95% of American adults have a latent varicella zoster virus (VZV) infection arising from a primary infection, varicella (chickenpox) [1]. About one in every five individuals with latent VZV will develop herpes zoster (shingles), a reactivation of the virus that typically presents as a painful vesicular rash within a dermatome [2]. While this rash resolves in several weeks, herpes zoster may produce additional complications including post herpetic neuralgia, ocular pathologies, myelitis, and encephalitis [2]. Approximately 10–20% of patients with shingles will have herpes zoster ophthalmicus, VZV reactivation in the ophthalmic division of the trigeminal nerve, which lies close to the cerebral arteries [3].

Multiple case studies have reported the occurrence of stroke after the development of shingles, but have not quantified the magnitude of risk associated with this viral reactivation [4–6]. Better understanding of this relationship is important to public health initiatives, since shingles is both common and preventable through vaccination [7, 8]. Since prior meta-analyses neither included more contemporary studies and have focused on cerebrovascular events to the exclusion of cardiac events, a more current synthesis of the existing literature is warranted [9–12]. In this review, we systematically describe and evaluate the literature examining the association between herpes zoster and the development of a variety of cerebrovascular and cardiac events and provide an overview of previous meta-analysis.

## Methods

### Data sources and searches

This review was performed in accordance with the Preferred Reporting Items for Systematic Reviews and Meta-Analyses (PRISMA) [13] (S1 Checklist). The protocol for this study was registered on the PROSPERO registry for systematic reviews (CRD42016045194) [14]. Searches on the PubMed^®^ (includes MEDLINE^®^ content) and Scopus^®^ (includes Embase^®^ content) electronic databases were performed in December 2016 to identify relevant articles. Text headings and medical subject heading (MeSH) terms used in the searches included herpes zoster, shingles, herpes zoster ophthalmicus, herpes zoster oticus, zoster sine herpete, herpes zoster oticus, stroke, transient ischemic attack, myocardial infarction, angina, cardiac death, coronary heart disease, coronary revascularization, and vascular diseases. This search strategy emphasized terms for the most prevalent cerebrovascular and cardiovascular events [15] (S1 File). We reviewed the reference lists of eligible articles and performed searches on Google Scholar and OAIster (database of the Open Archives Initiative) to identify additional research not indexed by the searched databases.

### Study selection

This review included studies that: (1) investigated the association between herpes zoster, or a subtype of herpes zoster, with cerebrovascular and cardiovascular events as defined in the primary studies and included stroke, transient ischemic attack, myocardial infarction, angina, coronary heart disease, cardiac death, coronary revascularization, and vascular diseases; (2) used a trial, cohort, case-control, case-crossover, or cross-sectional (with a temporal relationship established between exposure and outcome) design; (3) were written in English; (4) were published in a peer-review journal or as a conference paper; and (5) were published between January 1, 1960 and December 28, 2016. Excluded studies did not meet the inclusion criteria or (1) were a case-study/series, abstract (with no accompanying full text), review article, or cross-sectional study without the temporal relationship established between the exposure and outcome; or (2) examined primary VZV infection (i.e., chickenpox) as the exposure; or (3) restricted to patients less than 18 years of age; or (4) studied immunocompromised populations (e.g., HIV, cancer).

### Data extraction

One author (N.E.) reviewed all publication titles to eliminate articles that covered a subject not relevant to this review, described a primary exposure that was not herpes zoster or its subtypes, or used an immunocompromised, pediatric, or non-human sample. Two authors (N.E. and H.T.) reviewed abstracts of the remaining articles to determine inclusion for assessment of the full text. Discrepancies were resolved by group consensus and independent adjudication (R.G.) if consensus could not be reached. One author (N.E.) extracted data onto a standardized data collection form. Extracted information included study title and authors, year of publication, source of participants and sample size, mean age and gender composition of the study sample, type of herpes zoster studied (e.g., herpes zoster, herpes zoster ophthalmicus), cerebrovascular and cardiovascular event outcomes (e.g., stroke, transient ischemic attack, myocardial infarction, angina, coronary heart disease, cardiac death, coronary revascularization, and vascular diseases), and effect estimates with accompanying 95% confidence intervals. The extracted forms were reviewed (H.T.) for accuracy with the source paper.

### Risk of bias

To assess the quality of abstracted information, including the clarity of reporting study methods, error, and bias, we used questions adapted from the 32-item questionnaire developed by Downs and Black to rate patient intervention studies [16]. Following the approach of other systematic reviews [17–19], we removed items irrelevant to observational studies not studying an intervention as the primary exposure. Due to a lack of validation, we did not calculate a composite quality score but report scores in the appendix. Since the number of studies we included was low, we did not report the results of the Eggers test.

### Data synthesis and analysis

We performed qualitative and quantitative syntheses. We organized our findings according to type of herpes zoster, outcome (cerebrovascular or coronary event), and time examined between the onset of herpes related illness and our pre-defined disease outcomes. We considered meta-analysis when 2 or more studies could be pooled after evaluating the studies for clinical and statistical heterogeneity. Clinical heterogeneity was examined by evaluating for differences in study population, intervention or comparators. Statistical heterogeneity was estimated using the I^2^ statistic to evaluate both the random and fixed effects models. I^2^ values of 30–60% represented a moderate level of heterogeneity [20].

We conducted both random and fixed effects meta-analysis. We conducted random effects meta-analysis using the inverse variance method for pooled odds ratios (ORs). We used the fixed effects model when the number of studies was low. We assumed similarity between the Ors and other relative measures, such as relative risk, rate ratios, or hazard ratios because the cardiac events examined were relatively infrequent (< 1/10) events [21]. Where possible, we pooled adjusted Ors from the primary studies; otherwise, we used the unadjusted Ors with no correction for baseline differences or confounding. We assessed for publication bias using the Eggers test. All meta-analyses were conducted in Stats Direct.

Since prior studies have reviewed the association between herpes zoster and cerebrovascular events, but not cardiac events, and estimated pooled ORs, we summarized the findings of these studies forest plots to facilitate comparisons with our results.

## Results

**Fig 1:**
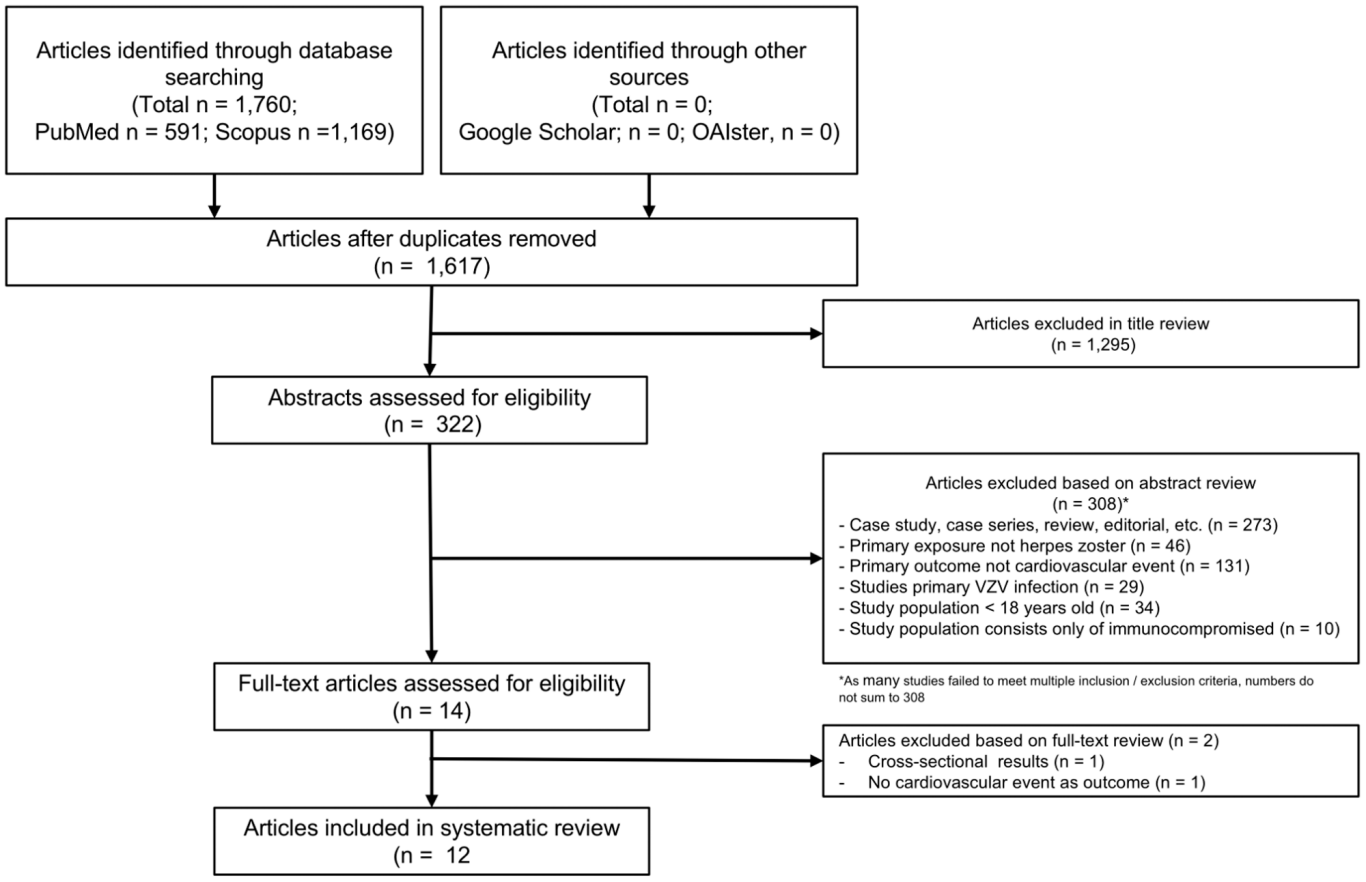
PRISMA flow chart describing the process for identifying included articles.

From the 1,617 unique articles initially identified in searches of electronic databases, 14 articles were selected for full-text review after title and abstract review as shown in the PRISMA flow sheet of included studies (Fig 1). Of these, twelve studies satisfied criteria for inclusion in this review (Table 1).

**Table 1:** Overview of included studies examining the association of herpes zoster and its subtypes with cardiovascular events.

Reference (Year)	Country	Study Design	Population Studied	Sample Size	Age Range (Years)	Sample Age (Years)	Follow Up Period (Years)	Herpes Zoster Type(s) Examined	Assessment of Exposure and Outcomes	Outcomes Examined
Breuer *et al*. (2014)	United Kingdom	Retrospective matched cohort	Patients of 464 primary care clinics (2002 to 2010)	319,803	≥ 18	MN: 57.8	24	HZ, HZO	READ, THIN codes	Stroke, TIA, MI
Kang *et al*. (2009)	Taiwan	Retrospective matched cohort	Random sample of National Health Insurance enrollees (1997 to 2001)	31,040	≥ 18	MN: 46.7	1	HZ, HZO	ICD-9-CM codes	Stroke
Kwon *et al*. (2016)	South Korea	Retrospective cohort	Random sample of National Health Insurance enrollees (2002–2013)	766,179	≥ 18	MN: 41.4	11	HZ	ICD-10 codes	Composite of Stroke and TIA
Langan *et al*. (2014)	United Kingdom	Self-controlled case-series	Patients receiving care at 625 primary care clinics (1987 to 2012)	6,584	≥ 18	MD: 77	25	HZ, HZO	CPRD, READ, ICD-10 codes	Stroke
Lin *et al*. (2010)	Taiwan	Retrospective matched cohort	National Health Insurance Service enrollees (2003 to 2005)	2,632	≥ 18	MN: 56.9	1	HZO	ICD-9-CM codes	Stroke
Minassian *et al*. (2016)	USA	Self-controlled case-series	Medicare enrollees 65 years and older (2006 to 2011)	42,954 (Stroke), 24,237 (MI)	≥ 65	MD: 81.1 (Stroke), 80.3 (MI)	6	HZ, HZO	ICD-9-CM codes, receipt of antiviral therapy (for HZ/HZO)	Ischemic stroke, MI
Schink *et al*. (2016)	Germany	Self-controlled case-series	Enrollees of four insurance providers (2004–2011)	124,462	≥ 0	MN: 71.3	1	HZ, HZO	ICD-10-GM codes	Stroke
Sreenivasan *et al*. (2013)	Denmark	Retrospective Cohort	All hospital and prescription users (1995 to 2008)	4,620,980	≥ 18	NR	13	HZ	Prescription records (HZ), ICD-10 codes (Stroke/TIA)	Composite of Stroke and TIA
Sundström *et al*. (2015)	Sweden	Retrospective Cohort	All residents of Västra Götaland County (2008–2010)	1.5 million	≥ 0	NR	1	HZ	ICD-10 Codes	Stroke
Wang *et al*. (2014)	Taiwan	Retrospective matched cohort	Enrollees of national insurance program (1999 to 2010)	289,790	≥ 0	NR	12	HZ	ICD-9-CM codes	ACS
Wu *et al*. (2015)	Taiwan	Retrospective matched cohort	Random sample of National Health Insurance enrollees (1998 to 2008)	97,415	≥ 20	MN: 46.4	10	HZ	ICD-9-CM codes	CAD
Yawn *et al*. (2016)	USA	Retrospective matched cohort	Residents of Olmsted country (MN) (1986 to 2010)	24,295	≥ 50	MN: 68.1	28	HZ	ICD-9-CM codes, medical record review	Stroke, MI

Abbreviations by alphabetic order: ACS: acute coronary syndromes, CAD: coronary artery disease (includes acute and subacute MI, angina pectoris), CM: Clinical Modification, CPRD: Clinical Practice Research Datalink (electronic database of patients receiving care at more than 625 UK primary care clinics), GM: German Modification, HZ: herpes zoster (type unspecified), HZO: herpes zoster ophthalmicus, ICD: International Classification of Diseases, MD: median, MI: myocardial infarction MN: mean, READ: clinical coding system used by United Kingdom General Practitioners, THIN: The Health Improvement Network (medical, prescription and demographic database on 3 million primary care patients in the UK), TIA: Transient Ischemic Attack

### Study characteristics

Table 1 presents an overview of the eight retrospective cohort studies [22–30] and three self-case controlled case series [31–33] included in this review. We included self-controlled case series since these studies have a design akin to that of an efficient cohort study that minimizes confounding and produces measures of association, unlike a traditional case series [34]. The studies sampled populations from six countries including enrollees of the national health insurance systems in Taiwan [23, 25, 28, 29] and South Korea [24], Danish hospital and prescription drug users [26], British primary care patients [22, 31], American Medicare enrollees 65 years and older [32], residents 50 years and older in Olmsted County (MN, US) [30], enrollees of four private insurers in Germany [33] and residents of Västra Götaland County, Sweden [27]. All included studies performed retrospective reviews of electronic medical record databases; no study prospectively followed patients.

As exposures, six of the included studies examined any form of herpes zoster (herpes zoster type unspecified, which includes herpes zoster ophthalmicus) [24, 26–30], five examined herpes zoster type unspecified with sub-group analyses for herpes zoster ophthalmicus [22, 23, 31–33], and one study only examined patients with herpes zoster ophthalmicus [25].

Included studies examined cerebrovascular and cardiovascular events over time periods ranging from 1 week to 24 years after onset of herpes zoster. The studies used a heterogeneous definition of cerebrovascular and cardiac events. Eleven of the included studies examined associations between herpes zoster type unspecified and the following: non-specified stroke [22, 23, 27, 30, 31, 33], ischemic stroke [32, 33], hemorrhagic stroke [33] [33], TIA [22], a composite of stroke and TIA [24, 26], myocardial infarction [22, 30, 32], acute coronary syndromes (e.g., acute myocardial infarction and unstable angina) [28], and incident coronary artery disease, including angina and myocardial infarction [29]. Among studies examining patients with herpes zoster ophthalmicus, five studies examined non-specified stroke [22, 23, 25, 31, 33], two studies examined ischemic stroke [32], one examined hemorrhagic stroke [33], and one examined myocardial infarction [32] as endpoints.

#### Quality of included studies

The details of the quality scores are presented in S2 File. The included cohort studies presented limited data on potential confounders, particularly on patient lifestyle practices that may lead to cerebrovascular or cardiovascular events [22–30]. While the self-controlled case series had limited measures of patient characteristics, their use of patients as their own controls adjusted for time-invariant confounders [31–33]. Besides items covered by the quality instrument, we observed that the included studies relied only on administrative codes to determine outcomes and/or pharmacy data to determine exposure status respectively, and were susceptible to misclassification of outcome and exposure. Only two studies used additional criteria, including medical record documentation [30] and prescription records [32], in combination with diagnostic codes, to validate exposure to herpes zoster. We also observed that the cohort studies did not account for the increased probability of finding statistically significant associations due to large sample sizes [22, 23, 25–32].

### Meta-analysis of herpes zoster and cerebrovascular events

In examining the relationships between herpes zoster, type unspecified, and cerebrovascular events, five studies found significantly positive associations with non-specific stroke [22, 23, 27, 30, 31, 33], two studies found associations with ischemic stroke [32, 33], one study with hemorrhagic stroke [33], one study with TIA [22], and two studies with a composite endpoint of stroke and TIA [24, 26].

**Fig 2:**
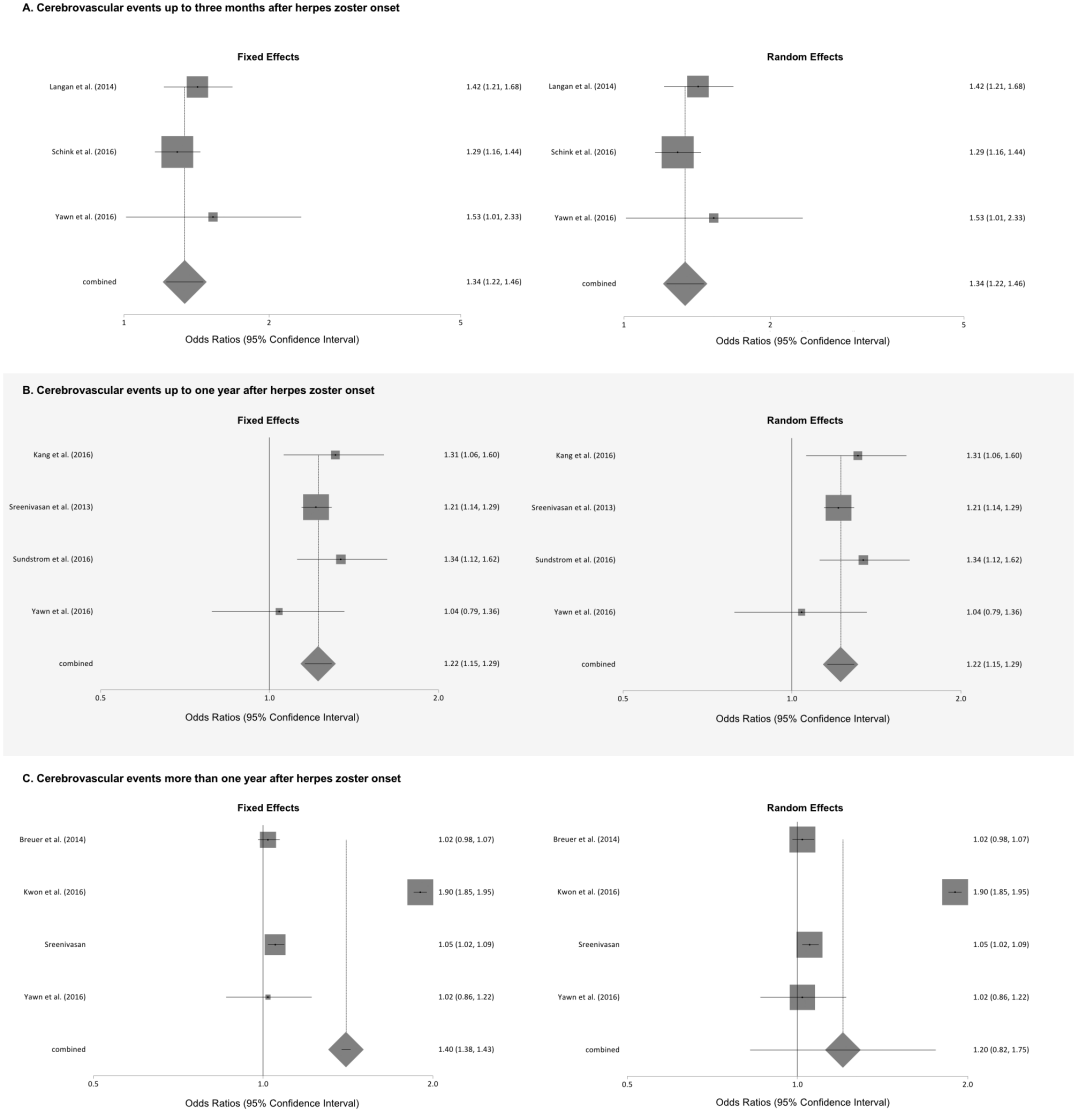
Meta-analyses of the association of herpes zoster, type unspecified, and cerebrovascular events stratified by length of follow-up.

The results of the meta-analyses of the association of herpes zoster, type unspecified, and cerebrovascular events stratified by the length of follow-up are shown in Fig 2. Meta-analyses found that patients with herpes zoster were at significantly increased odds of a cerebrovascular event within 3 months of onset using the random effects and fixed effects models (pooled Ors for fixed and random effects models 1.34, 95% CI: 1.22 to 1.46). There was no evidence for statistical heterogeneity (I^2^ = 0.0%) for the pooled results for cerebrovascular events. Similarly, the odds of a cerebrovascular event were significantly increased up to 1 year after herpes zoster onset (pooled Ors for fixed and random-effects models 1.22, 95% CI: 1.15 to 1.29), but with severe statistical heterogeneity (I^2^ = 99.7%). The odds of experiencing a cerebrovascular event over periods of time greater than 1 year after herpes zoster onset was significant in the fixed effects model (pooled OR 1.40, 95% CI: 1.38 to 1.43), but not the random effects model (pooled OR 1.20, 95% CI: 0.82 to 1.75), with severe statistical heterogeneity (I^2^ = 99.7%).

### Meta-analysis of herpes zoster and cardiac events

Overall, three studies found significantly positive associations between exposure to herpes zoster, type unspecified, with myocardial infarction within one week [32], three months [30], or twenty-four years after zoster onset [22], with acute coronary syndromes within 12 years after zoster onset [28], and with incident coronary artery disease within two and 10 years after zoster onset (Table 2) [29].

**Table 2:** Summary of measures of association between herpes zoster (type unspecified)* and cardiovascular events in reviewed studies.

Outcome	Reference	Follow Up Period After HZ	*n* Events / *n* with HZ	Adjustment Variables	Measure of Association**	Adjusted Association Size (95% CI)
Cerebrovascular Events						
Stroke (Non-Specified)	Langan *et al*.	3 months	149**	AGE	IR	1.42 (1.21 to 1.68)
	Schink *et al*.	3 months	352**	AGE	IRR	1.29 (1.16 to 1.44)
	Yawn *et al*.	3 months	33 / 4,478	AGE, ARTHM, VASC	OR	1.53 (1.01 to 2.33)
	Yawn *et al*.	6 months	46 / NR	AGE, HTN, VASC	OR	1.28 (0.91 to 1.80)
	Kang *et al*.	1 year	133 / 7,760	AGE, SEX, HTN, DM, CHD, HYPLIP, RENAL, AF, HF, VALV, CRTD, INC, URBN, GEO	HR	1.31 (1.06 to 1.60)
	Sundström *et al*.	1 year	111 / 13,269	AGE, SEX	IRR	1.34 (1.12 to 1.62)
	Yawn *et al*.	1 year	71 / NR	AGE, SEX, HTN, DYSLIP, CHD, VASC	OR	1.04 (0.79 to 1.36)
	Yawn *et al*.	3 years	176 / 4,151	AGE, SEX, HTN, DYSLIP, CHD, VASC, DEP	OR	1.02 (0.86 to 1.22)
	Breuer *et al*	24 years	2,727 / 106,601	AGE, SEX, BMI, SMK, HYPLIP, HTN, DM, CHD, AF, PVD, CRTD, VALV	HR	1.02 (0.98 to 1.07)
Ischemic Stroke	Minassian *et al*.	1 week	499**	AGE	IR	2.37 (2.17 to 2.59)
	Schink *et al*.	3 months	310**	AGE	IRR	1.27 (1.13 to 1.42)
Hemorrhagic Stroke	Schink *et al*.	3 months	42**	AGE	IRR	1.53 (1.11 to 2.11)
TIA	Breuer *et al*.	24 years	2,275 / 106,601	AGE, SEX, BMI, SMK, HYPLIP, HTN, DM, CHD, AF, PVD, CRTD, VALV	HR	1.15 (1.09 to 1.21)
Stroke and TIA	Sreenivasan *et al*.	2 weeks	83 / 117,926	AGE, SEX, SN	IRR	2.27 (1.83 to 2.82)
	Sreenivasan *et al*.	1 year	4,876 / 117,926	AGE, SEX, SN	IRR	1.21 (1.14 to 1.29)
	Sreenivasan *et al*.	1 year				1.05 (1.02 to 1.09)
	Kwon *et al*.	11 years	5,069 / 77,781	AGE, SEX, HTN, HYPLIP, CHD, DM, HF, PVD, AF, RENAL, VALV	IRR	1.90 (1.85 to 1.95)

*Cases of herpes zoster ophthalmicus were included in all cases of the exposures,

** Comparison of patients with herpes zoster to those without (referent group)

**Abbreviations (Alphabetically):** AF: atrial fibrillation, ARTHM: arrhythmia, BMI: body mass index, CLIN: frequency of clinical visits, CNCR: cancer, CRTD: carotid disease, CHD: coronary heart disease, COPD: chronic obstructive pulmonary disease, CVD: cerebral vascular disease, DEP: depression, DM: diabetes mellitus, GEO: geographical region, HF: heart failure: HR: hazard ratio, HYPLIP: hyperlipidemia, HTN: hypertension, HZ: Herpes Zoster, INC: income, IR: incidence ratio, IRR: incidence rate ratio, MEDS: medication use, NR: not reported, OCC: occupation, PVD: peripheral vascular disease, RENAL: renal disease, SCCS: self-controlled case series, SN: season, SMK: smoking statin, URBN: urbanization level of patient’s area of residence, VALV: valvular disease

**Fig 3:**
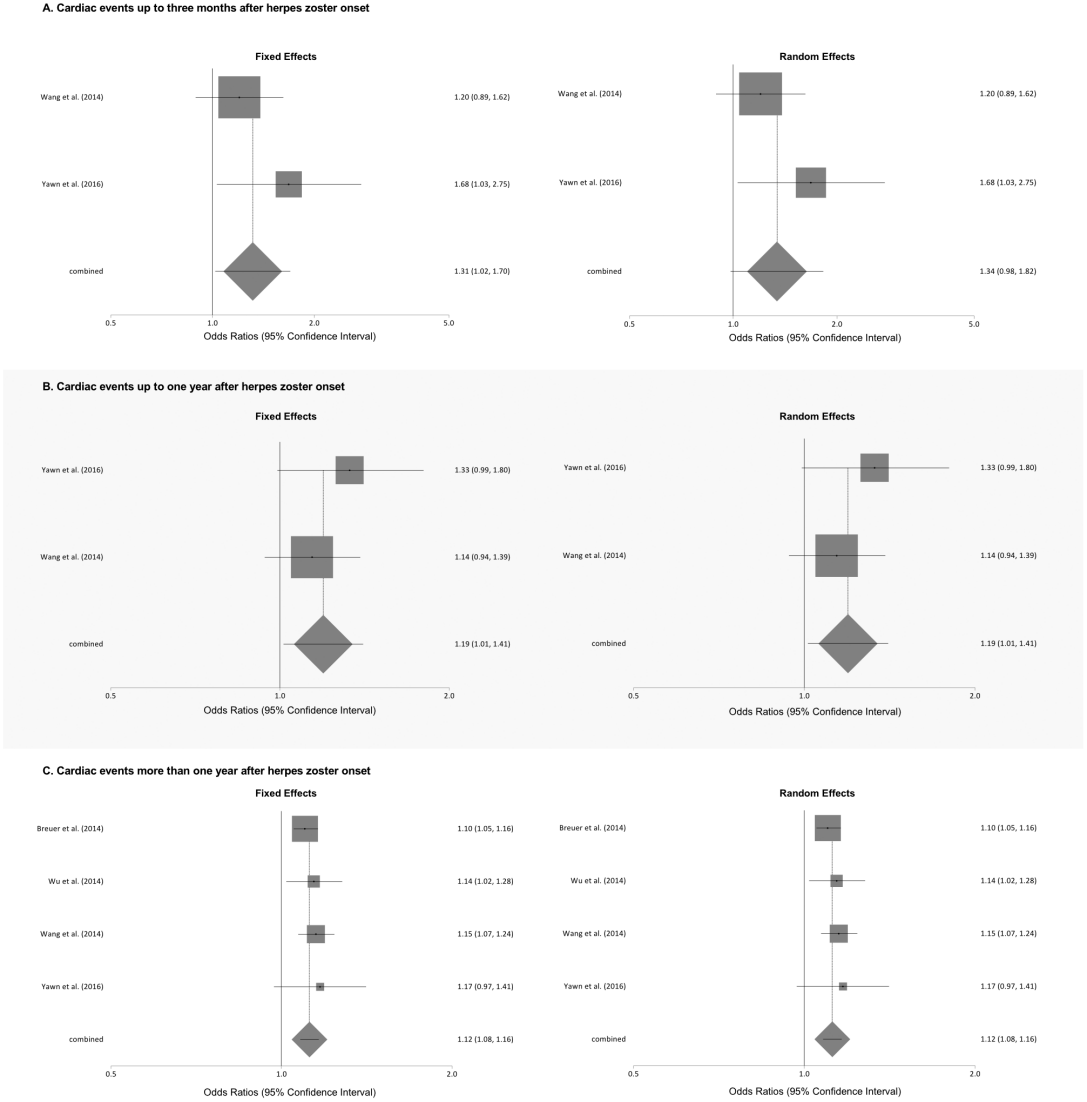
Meta-analyses of the association of herpes zoster, type unspecified, and cardiac events stratified by length of follow-up.

Meta-analyses of the association of herpes zoster, type unspecified, and various cardiac events stratified by length of follow-up is shown in Fig 3. Patients with herpes zoster were at significantly increased odds of a cardiac event at 3 months after onset using the fixed effects model (pooled OR 1.31, 95% CI: 1.02 to 1.70), but not the random effects model (pooled OR 1.34. 95% CI 0.98 to 1.82). We found a low level of statistical heterogeneity (I^2^ = 23.9%) for the pooled results for myocardial infarction. The odds of a cardiac event up to 1 year after onset were significantly higher using the fixed and random effects model (both pooled Ors 1.19, 95% CI: 1.01 to 1.41). We found no evidence of statistical heterogeneity (I^2^ = 0.0%). Likewise, the odds of a cardiac event over periods of greater than 1 year after zoster onset were significantly higher using both the fixed and random effects models (both pooled Ors 1.12, 95% CI: 1.08 to 1.16). We found no evidence of statistical heterogeneity (I^2^ = 0.0%).

### Meta-analysis of herpes zoster ophthalmicus and cerebrovascular events

**Fig 4:**
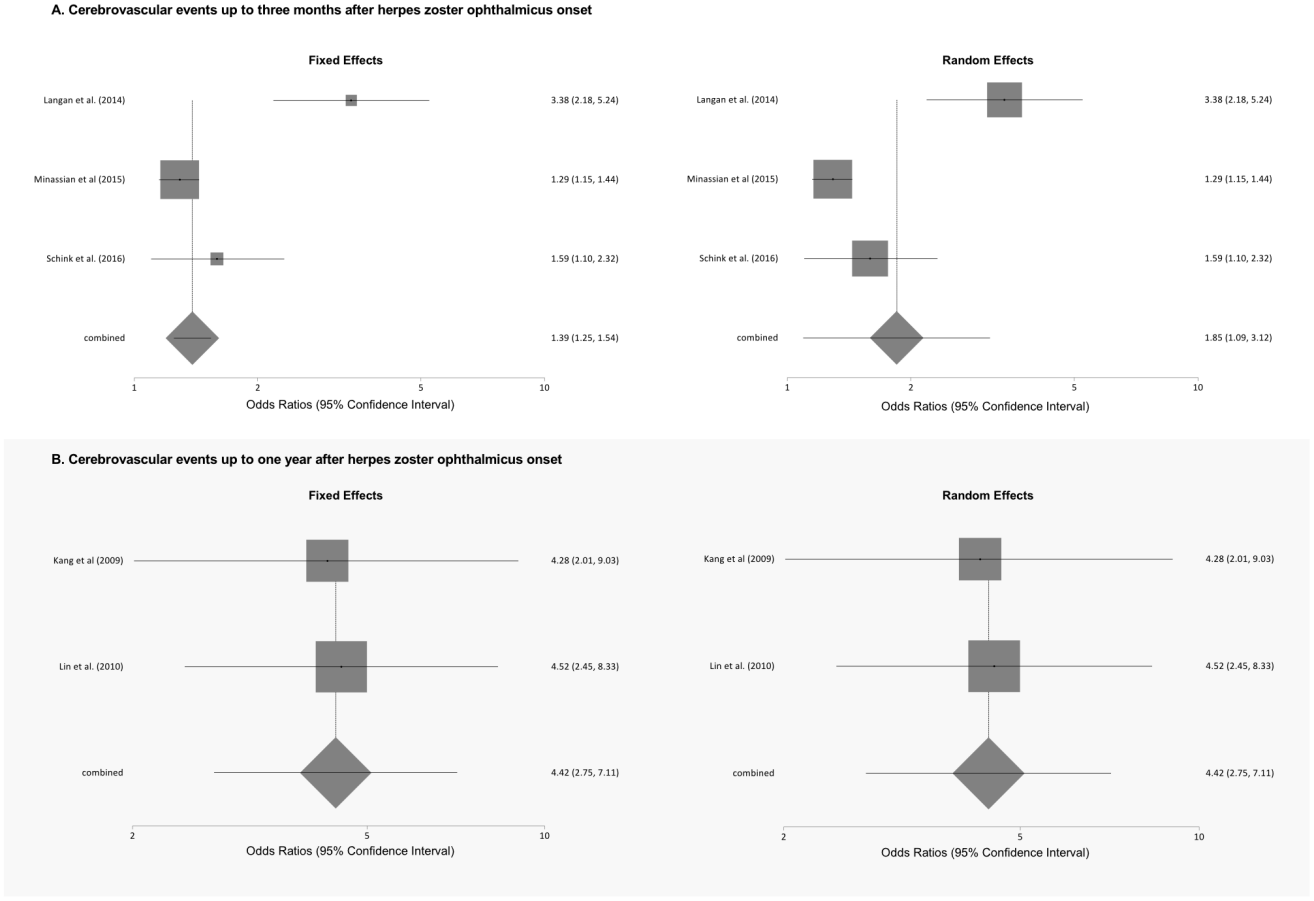
Meta-analyses of the association of herpes zoster ophthalmicus and cerebrovascular events stratified by length of follow-up.

Exposure to herpes zoster ophthalmicus was statistically significantly associated with development of non-specific stroke over time periods of up to one year in four studies [23, 25, 31, 33], with the development of ischemic stroke over periods up to three months in two studies [32, 33], but not with hemorrhagic stroke [33] (Table 3). Meta-analyses of the association of herpes zoster ophthalmicus and cerebrovascular events stratified by length of follow up are shown in Fig 4. Compared with unexposed individuals, patients with herpes zoster ophthalmicus were at significantly increased odds of cerebrovascular events within 3 months of illness onset in meta-analyses using fixed-effects (pooled OR 1.39, 95% CI: 1.25 to 1.54) and random effects (pooled OR 1.85, 95% confidence interval 1.09 to 3.12) models. We found a high level of statistical heterogeneity (I^2^ = 88.9%) for the pooled results for cerebrovascular events. Similarly, patients exposed to herpes zoster ophthalmicus had an increased odds of cerebrovascular events within 1 year of onset in meta-analyses using both the fixed and random effects models (both pooled ORs 4.42, 95% confidence interval 2.75 to 7.11), without any evidence of statistical heterogeneity (I^2^ = 0%).

**Table 3:** Summary of measures of association between herpes zoster (type unspecified)* and cardiac events in reviewed studies.

Outcome	Reference	Follow Up Period After HZ	*n* Events / *n* with HZ	Adjustment Variables	Measure of Association**	Adjusted Association Size (95% CI)
Cardiac Events						
Myocardial Infarction	Minassian *et al*	1 week	213**	AGE	IR	1.68 (1.47 to 1.92)
	Yawn *et al*.	3 months	24 / 4,405	AGE, CHD	OR	1.68 (1.03 to 2.75)
	Yawn *et al*.	6 months	35 / NR	AGE, CHD, DM	OR	1.44 (0.97 to 2.15)
	Yawn *et al*.	1 year	61 / NR	AGE, SEX, CHD, DM, DEP	OR	1.33 (0.99 to 1.80)
	Yawn *et al*.	3 year	154 / 4,102	AGE, SEX, HTN, CHD, DM	OR	1.17 (0.97 to 1.41)
	Breuer *et al*.	24 years	2,762 / 106,601	AGE, SEX, BMI, SMK, DYSLIP, HTN, DM, CHD, AF, PVD, CRTD, VALV	HR	1.10 (1.05 to 1.16)
Acute Coronary Syndromes	Wang *et al*.	3 months	58 / 57,958	AGE, SEX, URBN, INC, OCPTN, CLIN, HTN, DM, HYPLIP, CVD, COPD, RENAL, CNCR, MEDS	HR	1.20 (0.89 to 1.62)
	Wang *et al*.	1 year	193 / 57,958	AGE, SEX, URBN, INC, OCPTN, CLIN, HTN, DM, HYPLIP, CVD, COPD, RENAL, CNCR, MEDS	HR	1.14 (0.94 to 1.39)
	Wang *et al*.	12 years	860 / 57,958	AGE, SEX, URBN, INC, OCPTN, CLIN, HTN, DM, HYPLIP, CVD, COPD, RENAL, CNCR, MEDS	HR	1.15 (1.07 to 1.24)
Coronary Artery Disease	Wu *et al*.	2 years	388 / 19,483	AGE, SEX, DM, HTN, HYPLIP	HR	1.14 (1.02 to 1.28)
	Wu *et al*.	10 years	1,057 / 19,483	AGE, SEX, DM, HTN, HYPLIP	HR	1.11 (1.04 to 1.19)

*Cases of herpes zoster ophthalmicus were included in all cases of the exposures,

** Comparison of patients with herpes zoster to those without (referent group)

**Abbreviations (Alphabetically):** AF: atrial fibrillation, ARTHM: arrhythmia, BMI: body mass index, CLIN: frequency of clinical visits, CNCR: cancer, CRTD: carotid disease, CHD: coronary heart disease, COPD: chronic obstructive pulmonary disease, CVD: cerebral vascular disease, DEP: depression, DM: diabetes mellitus, GEO: geographical region, HF: heart failure: HR: hazard ratio, HYPLIP: hyperlipidemia, HTN: hypertension, HZ: Herpes Zoster, INC: income, IR: incidence ratio, IRR: incidence rate ratio, MEDS: medication use, NR: not reported, OCC: occupation, PVD: peripheral vascular disease, RENAL: renal disease, SCCS: self-controlled case series, SN: season, SMK: smoking statin, URBN: urbanization level of patient’s area of residence, VALV: valvular disease

### Herpes zoster ophthalmicus and cardiac events

The one study to examine the association between herpes zoster ophthalmicus and acute cardiac events showed that the relative frequency of myocardial infarction was significantly higher in the first week after disease onset (Table 4) [32].

**Table 4:** Summary of measures of association between herpes zoster ophthalmicus and cardiovascular events in reviewed studies.

Outcome	Reference	Follow Up Period After HZO	*n* Events / *n* with HZ	Adjustment Variables	Measure of Association*	Adjusted Association Size (95% CI)
**Cerebrovascular Events**						
Stroke (Non-Specified)	Schink *et al*.	3 months	31*	AGE	IRR	1.59 (1.10 to 2.32)
	Langan *et al*.	4 months	22*	AGE	IR	3.38 (2.18 to 5.24)
	Kang *et al*.	1 year	7 / 120	AGE, SEX, HTN, DM, CHD, HYPLYP, RENAL, AF, HF, VALV, CRTD, INC, URBN, GEO	HR	4.28 (2.01 to 9.03)
	Lin *et al*.	1 year	53 / 658	AGE, SEX, HTN, HYPLIP, DM, CHD, RHD, MEDS	HR	4.52 (2.45 to 8.33)
	Breuer *et al*.	24 years	68 / 1,710	AGE, SEX, BMI, SMK, DYSLIP, HTN, DM, CHD, AF, PVD, CRTD, VALV	HR	1.03 (0.77 to 1.39)
Ischemic Stroke	Minassian *et al*.	1 week	93*	AGE	IR	2.73 (2.22 to 3.35)
	Minassian *et al*.	3 months	326*	AGE	IR	1.29 (1.15 to 1.44)
	Schink *et al*.	3 months	27*	AGE	IRR	1.57 (1.05 to 2.35)
Hemorrhagic Stroke	Schink *et al*.	3 months	4*	AGE	IRR	1.82 (0.62 to 5.37)
**Coronary Events**						
Myocardial Infarction	Minassian *et al*.	1 week	43 (SCCS)	AGE	IR	2.06 (1.52 to 2.79)

* Comparison of patients with herpes zoster to those without (referent group)

**Abbreviations (Alphabetically):** AF: atrial fibrillation, BMI: body mass index, CLIN: frequency of clinical visits, CNCR: cancer, CRTD: carotid disease, CHD: coronary heart disease, COPD: chronic obstructive pulmonary disease, CVD: cerebral vascular disease, DEP: depression, DM: diabetes mellitus, GEO: geographical region, HF: heart failure: HR: hazard ratio, HYPLIP: hyperlipidemia, HTN: hypertension, HZ: Herpes Zoster, INC: income, IR: incidence ratio, IRR: incidence rate ratio, MEDS: medication use, NR: not reported, OCC: occupation, PVD: peripheral vascular disease, RENAL: renal disease, SSN: season, SMK: smoking statin, URBN: urbanization level of patient’s area of residence, VALV: valvular disease

### Overview of previous systematic reviews

**Fig 5:**
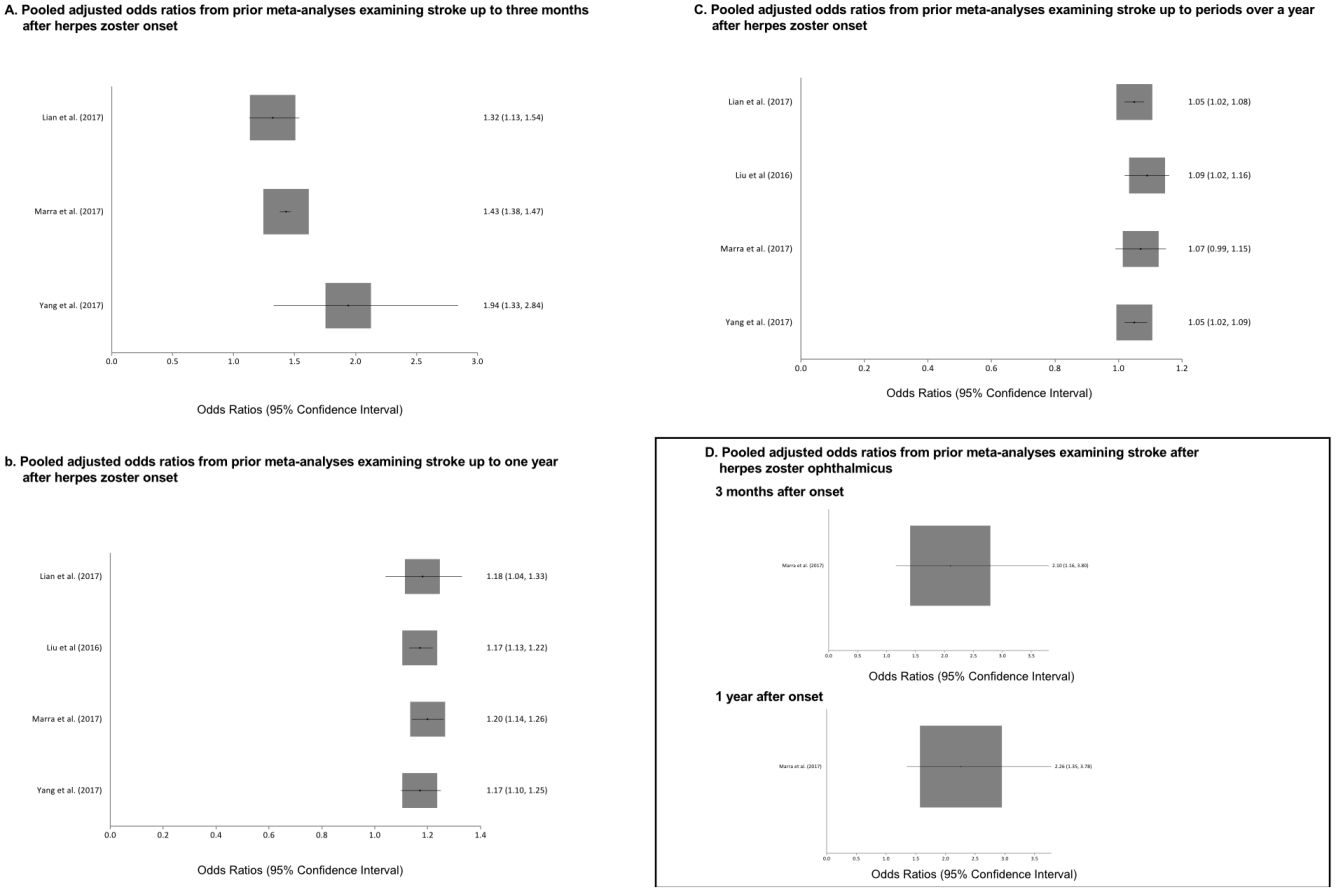
Results of prior meta-analyses examining the association between herpes zoster and herpes zoster ophthalmicus with stroke.

The Forest plots from previous meta-analysis examining the association between herpes zoster, type unspecified and herpes zoster ophthalmicus with stroke are shown in Fig 5. All meta-analyses show a consistently positive relationship between herpes zoster and herpes zoster ophthalmicus with stroke.

## Discussion

Our systematic review and meta-analyses of twelve published epidemiological studies identified associations with exposure to herpes zoster with increased odds of 20 to 40% and 10 to 30% of experiencing a cerebrovascular and cardiovascular event, respectively, over periods of 3 months to over a year after onset. Similarly, compared with healthy controls, those exposed to herpes zoster ophthalmicus may have twice to four times the odds of experiencing a cerebrovascular event, a relatively larger magnitude of effect, over periods of up to a year. To the best of our knowledge, this is the first meta-analysis suggesting an association between herpes zoster and cardiac events. These findings must be interpreted in the context of relatively small magnitude of association, however, and the limited quality of included studies.

Our results also confirm the positive relationship between herpes zoster and herpes zoster ophthalmicus with stroke found in prior meta-analyses [9–12]. Similar to our findings, these four meta-analyses also found that the magnitude of association between herpes zoster and stroke also declined over longer periods of follow-up. The one meta-analysis that examined herpes zoster ophthalmicus did not show a substantial change in the pooled odds ratio of experiencing a stroke at 3 months versus 1 year after herpes onset [11]. In contrast, our study found a greater magnitude of association between herpes zoster ophthalmicus and cerebrovascular events at one year compared to 3 months after herpes onset. This discrepancy may result from limiting our analysis to observation periods soon after the onset of herpes zoster ophthalmicus. In contrast, the prior meta-analysis incorporated measures of association from observation periods starting months after the diagnosis of herpes zoster ophthalmicus. Our analyses of stroke with herpes zoster and herpes ophthalmicus also incorporated findings from one or more contemporary publications that were not included in prior meta-analyses [33].

While recognizing that our results do not definitely prove herpes zoster to be a cause of cardiovascular events, several plausible mechanisms exist to account for our findings. The development of herpes zoster may lead to migration of VZV from the neurons to the cerebral and coronary vasculature. This may lead to a local inflammatory response, causing vessel occlusion and ultimately ischemia [35]. A case series of seven adults with symptoms of herpes zoster and stroke or TIA found that these patients had cerebrospinal fluid samples that were positive for VZV DNA and anti-VZV antibodies [36][35]. Arterial biopsies from patients with shingles and stroke showed the presence of intranuclear Cowdry A inclusions consistent with VZV [37]. Patients with herpes zoster ophthalmicus could be susceptible to a cerebrovascular event due to the proximity of the trigeminal ganglion to the cerebral arteries; our meta-analytic results showed a more than two-fold increased odds of cerebrovascular events among patients with a history of herpes zoster ophthalmicus. Herpes zoster could also produce systemic inflammation, autoimmune responses, or hemodynamic changes leading to cardiovascular events [22, 32, 38].

Moreover, the association between herpes zoster and cardiovascular events could result from both diseases sharing similar precipitating factors. For instance, illness and psychological stress have been separately identified as independent triggers of herpes zoster and acute myocardial infarction [39, 40]. If such common factors were the cause for the observed associations, we would have expected that results from the reviewed self-controlled case series would have been closer to the null hypothesis [31–33]. Future studies in this area would benefit from the assessment of, and accounting for, common causes and/or triggers of herpes zoster and acute cardiac events.

Since most of the evidence included in this review came from retrospective studies of claims data, additional study is warranted to confirm and better characterize the magnitude and timing of potential cardiovascular complications following herpes zoster. While many of these studies had the benefit of using large cohorts that were representative of general populations, past reviews of medical records suggest that some misclassification of patients with diagnostic codes for stroke, myocardial infarction, and herpes zoster can occur [41–43]. Subsequent studies may be improved by applying more stringent criteria for the diagnosis of herpes zoster (i.e., viral PCR or direct fluorescence antigen testing), stroke (i.e., computed tomography or magnetic resonance imaging), and acute cardiac events (elevated cardiac biomarkers or serial electrocardiographic changes) [44–46]. Moreover, with the exception of herpes zoster ophthalmicus, claims data may lack information on the dermatomal distribution of the presenting zoster. Conceivably, the risk of cardiovascular events may depend on whether the herpes zoster outbreak occurs in dermatomes that share innervation with the coronary and cerebral arteries.

Additional studies of cardiovascular outcomes among patients receiving vaccination against, as well as antiviral therapy for, herpes zoster could provide a better understanding of herpes zoster as a possible risk factor for cardiovascular diseases. For instance, data that suggests that those receiving vaccinations against influenza have decreased rates of acute coronary syndromes have provided evidence for the pathogen as being a trigger for cardiac events [47, 48]. Future cohort studies or (preferably) randomized trials examining the occurrence of cardiovascular events among those receiving the shingles vaccine could lead to better clarification of the possible relationship between herpes zoster and the outcomes suggested by our results.

Although the Food and Drug administration has approved use of the shingles vaccine in person 50 years and older, the Centers for Disease Control (CDC) only recommends vaccination against herpes zoster for all adults 60 years and older [49]. The CDC does not have a recommendation for the use of vaccine for those in the 50 to 59 year age group. The lack of recommendations for this age group is based on the low incidence of herpes zoster in the age group, limited duration of the efficacy of the vaccine, and lack of evidence for cost-effectiveness [49, 50]. However, age-stratified sub-group analyses from several of the studies included in this review suggest that patients younger than 60 years may have a heightened risk of cardiovascular events following herpes zoster [22, 24, 26–28]. Since published trials on herpes zoster vaccine lack data on the frequency of cardiac events in populations both less than and greater than 60 years old [51, 52], further research is required for the potential for vaccination against herpes zoster to reduce cardiovascular events, possibly in age-groups that do not traditionally receive the vaccine.

While our results are insufficient to suggest changes in clinical practice, consideration is warranted on research for potential management strategies for patients with herpes zoster if there is the potential that these individuals may have a greater likelihood of future cardiovascular events. As historical results suggest that the public generally possesses limited knowledge about the acute symptoms of stroke and myocardial infarction, future work could examine educating patients with herpes zoster about the symptoms of acute coronary disease [53, 54].

Moreover, future research may consider the effects of antiviral therapy on the subsequent risk of cardiovascular events among patients with herpes zoster. In general, recommendations suggest treatment of herpes zoster with a guanosine analogue, such as acyclovir, within 72 hours of the development of acute symptoms or in the presence of other criteria, such as being older or immunosuppressed [44]. Since such guidelines were developed with the intention of managing pain and neurologic complications from herpes zoster, treatment for the prevention of cardiovascular events may be different. Limited observational analyses suggest that patients receiving antiviral therapy after the onset of herpes zoster may have decreased risks of experiencing subsequent cardiovascular events[25, 28, 31] but investigations using data from randomized, controlled studies would likely be required for more definitive evidence.

We acknowledge several limitations of this review which primarily reflect the quality of reported data. We could not conduct time to event analysis in the absence of individual level data. We may have missed some studies that were not published in English. Positive-results bias may have prevented the publication of studies that did not find an association between herpes zoster and selected cardiovascular events. Four studies included in this review sampled enrollees in a Taiwanese health insurance program; while each of those studies sampled slightly different populations, the results of these studies could potentially have a similar error or biases that would not occur from studies that used more diverse data sources. All of the included studies utilized a retrospective design that ascertained exposure and outcomes through diagnosis codes—only two studies confirmed the diagnosis of herpes zoster with additional record review of microbiology data [30,32]. Moreover, these studies could not account for patients who developed herpes zoster and/or a cardiovascular event and did not seek medical treatment.

## Conclusions

Our systematic review and meta-analysis suggests that the occurrence of cerebrovascular and cardiac events increases following the development of herpes zoster, although the magnitude of the effect is small and the quality of evidence is limited. Further studies are needed to characterize this relationship and to determine whether more aggressive prevention of herpes zoster through vaccination could reduce the burden of cerebrovascular and cardiovascular disease associated with this infection. Future studies should also identify optimal approaches for the treatment and surveillance of patients with herpes zoster to mitigate their possibly heightened risk of cerebrovascular and cardiovascular events.

## Supporting information

S1 Checklist PRISMA checklist. (PDF)

S1 File Appendix I: Strategy of electronic s20167earches. (PDF)

S2 File Appendix II: Quality scores for studies included in systematic review and meta-analysis. (PDF)
