# Supplementary material for: A systematic review and meta-analysis on herpes zoster and the risk of cardiac and cerebrovascular events
Source: PLoS One. 2017 Jul 27;12(7):e0181565. doi: 10.1371/journal.pone.0181565 (PMC5531458; doi:10.1371/journal.pone.0181565)
Supplement: S1 File — (PDF) [file pone.0181565.s002.pdf]

Appendix 1: Electron Search Strategies

Source: PubMed

Dates Searched: Jan 1 1960 to Dec 28 2016

Search Term:
("Herpes Zoster"[Mesh] OR “herpes zoster” OR “herpes zoster ophthalmicus” OR “herpes zoster oticus” OR “herpete sine zoster” OR “shingles”) AND ("Stroke"[Mesh] OR "Ischemic Attack, Transient"[Mesh] OR "Myocardial Infarction"[Mesh] OR "Angina Pectoris"[Mesh] OR "Angina, Stable"[Mesh] OR "Death, Sudden, Cardiac"[Mesh] OR "Heart Diseases"[Mesh] OR "Vascular Diseases"[Mesh] OR stroke[tiab] OR “transient ischemic attack” OR tia[tiab] OR “myocardial infarction” OR angina[tiab] OR “cardiac death” OR “coronary heart disease” OR revascularization[tiab]) AND english[Language] AND ("1960/01/01"[Date - Publication] : "2016/12/28"[Date - Publication])

Source: SCOPUS (Embase)

Dates Searched: Jan 1 1960 to Dec 28 2016

Search Term:
({herpes zoster} OR {herpes zoster ophthalmicus} OR {herpes zoster oticus} OR {herpete sine zoster} OR {shingles} AND {heart diseases} OR {vascular diseases} OR "stroke" OR {transient ischemic attack} OR "tia" OR {myocardial infarction} OR "angina" OR {cardiac death} OR {coronary heart disease} OR "revascularization") AND (LIMIT-TO(LANGUAGE,"English")) AND (LIMIT-TO(DOCTYPE,"ar") OR LIMIT-TO(DOCTYPE,"cp") OR LIMIT-TO(DOCTYPE,"ip")) AND PUBYEAR > 1959
