# Supplementary material for: A systematic review and meta-analysis on herpes zoster and the risk of cardiac and cerebrovascular events
Source: PLoS One. 2017 Jul 27;12(7):e0181565. doi: 10.1371/journal.pone.0181565 (PMC5531458; doi:10.1371/journal.pone.0181565)
Supplement: S2 File — (PDF) [file pone.0181565.s003.pdf]

Appendix: Quality scores for studies included in systematic review and meta-analysis

Reference (year)	1 Is the hypothesis/ aim/objective of the study clearly described? Yes = 1 No = 0	2 Are the main outcomes to be measured clearly described in the introduction or methods section? Yes = 1 No = 0	3 Are the characteristics of the patients included in the study clearly described? Yes = 1 No = 0	4 Are the distributions of principal confounders in each group of subjects to be compared clearly described? Yes = 2 Part = 1 No = 0	5 Are the main findings of the study clearly described? Yes = 1 No = 0	6 Does the study provide estimates of the random variability in the data for the main outcomes? Yes = 1 No = 0	7 Have the characteristics of patients lost to follow-up been described? Yes = 1 No = 0	8 Have actual probability values been reported (e.g., 0.035 rather than <0.05) for the main outcomes except where the probability value is less than 0.001? Yes = 1 No = 0
Breuer *et al.* (2014)	1	1	1	1	1	1	NA	1
Kang *et al.* (2009)	1	0	1	1	1	1	NA	1
Kwon *et al.* (2016)	1	1	1	1	1	1	NA	1
Langan *et al.* (2014)	1	1	0	2	1	1	NA	1
Lin *et al.* (2010)	1	1	1	1	1	1	NA	1
Minassian *et al.* (2016)	1	1	1	1	1	1	NA	0
Schink *et al.* (2016)	1	1	1	1	1	1	NA	NA
Sreenivasan *et al.* (2013)	1	1	1	0	1	1	NA	1
Sundström *et al.* (2015)	1	0	1	0	1	1	NA	1
Wang *et al.* (2014)	1	1	1	1	1	1	NA	1
Wu *et al.* (2015)	1	1	1	1	1	1	NA	1
Yawn *et al.* (2016)	1	1	1	1	1	1	NA	1

Reference (year)	9 Were the subjects asked to participate in the study representative of the entire population from which they were recruited? Yes = 1 No = 0 Unable to determine = 0	10 Were those subjects who were prepared to participate representative of the entire population from which they were recruited? Yes = 1 No = 0 Unable to determine = 0	11 If any of the results of the study were based on "data dredging", was this made clear? Yes = 1 No = 0 Unable to determine = 0	12 In trials and cohort studies, do the analyses adjust for different lengths of follow-up of patients? Yes = 1 No = 0 Unable to determine = 0	13 Were the statistical tests used to assess the main outcomes appropriate? Yes = 1 No = 0 Unable to determine = 0	14 Were the main outcome measures used accurate (valid and reliable)? Yes = 1 No = 0 Unable to determine = 0	15 Was there adequate adjustment for confounding in the analyses from which the main findings were drawn? Yes = 1 No = 0 Unable to determine = 0	16 Were losses of patients to follow-up taken into account? Yes = 1 No = 0 Unable to determine = 0
Breuer *et al.* (2014)	1	1	1	1	0	1	1	1
Kang *et al.* (2009)	1	1	1	1	1	1	1	1
Kwon *et al.* (2016)	1	1	0	1	1	1	0	1
Langan *et al.* (2014)	1	1	1	1	1	1	1	1
Lin *et al.* (2010)	1	1	1	1	1	1	1	1
Minassian *et al.* (2016)	1	1	1	1	1	1	1	1
Schink *et al.* (2016)	1	1	1	1	1	1	1	1
Sreenivasan *et al.* (2013)	1	1	1	1	1	1	0	1
Sundström *et al.* (2015)	1	1	1	1	1	1	0	1
Wang *et al.* (2014)	1	1	1	1	1	1	0	1
Wu *et al.* (2015)	1	1	1	1	1	1	0	1
Yawn *et al.* (2016)	1	1	1	1	0	1	1	1

Reference (year)	**17**<br>Adequate sample size<br>Yes = 1<br>No = 0
Breuer *et al.* (2014)	1
Kang *et al.* (2009)	1
Kwon *et al.* (2016)	1
Langan *et al.* (2014)	1
Lin *et al.* (2010)	1
Minassian *et al.* (2016)	1
Schink *et al.* (2016)	1
Sreenivasan *et al.* (2013)	1
Sundström *et al.* (2015)	1
Wang *et al.* (2014)	1
Wu *et al.* (2015)	1
Yawn *et al.* (2016)	1
